# Differential Effects of Three Nutritional Supplements on the Nutrient Intake of Pregnant Women Enrolled in a Conditional Cash Transfer Program in Mexico: A Cluster Randomized Trial

**DOI:** 10.3390/nu14153003

**Published:** 2022-07-22

**Authors:** Fabiola Mejía-Rodríguez, Amado D. Quezada-Sánchez, Ignacio Méndez Gómez-Humarán, Raquel García-Feregrino, Armando García-Guerra, Ana Cecilia Fernández-Gaxiola, Lynnette M. Neufeld

**Affiliations:** 1 Centro de Investigación en Nutrición y Salud, Instituto Nacional de Salud Pública (INSP), Universidad N-655, Colonia Santa María Ahuacatitlán, Cerrada Los Pinos y Caminera, Cuernavaca 62100, Mexico; garciaf@insp.mx (A.G.-G.); anafdezg@hotmail.com (A.C.F.-G.); 2 Centro de Investigación en Evaluación y Encuestas, Instituto Nacional de Salud Pública (INSP), Universidad N-655, Colonia Santa María Ahuacatitlán, Cerrada Los Pinos y Caminera, Cuernavaca 62100, Mexico; amado.quezada@insp.mx (A.D.Q.-S.); fraquel@insp.mx (R.G.-F.); 3 Centro de Investigación de Matemáticas, Unidad Aguascalientes, Aguascalientes 20259, Mexico; imendez@cimat.mx; 4 Food and Agriculture Organization of the United Nations (FAO), 00153 Rome, Italy; lynnette.neufeld@fao.org

**Keywords:** women, micronutrient, fortified food

## Abstract

Supplementation in malnourished pregnant women should not displace natural healthy foods. Objective: To estimate the differential effects of three nutritional supplements on macro- and micronutrient intake of pregnant women beneficiaries of the conditional cash transfer program Prospera (CCT-POP). Methods: Prospective cluster randomized trial. Communities were randomly assigned to receive a fortified beverage (Beverage), micronutrient tablets (Tablets), or micronutrient powder (MNP). Pregnant women (at <25 weeks) were recruited. The food frequency questionnaire was applied at 25 and 37 weeks of pregnancy and at one and three months postpartum (mpp). Differential effects of the three supplements on the median change in nutrient intake from baseline to each follow-up stage were estimated. Results: Median change in protein intake from dietary and supplement sources were significantly lower for MNP and Tablets than for Beverages (baseline to 37 w: −7.80 ± 2.90 and −11.54 ± 3.00, respectively; baseline to 1 mpp: −7.34 ± 2.90 for MNP, *p* < 0.001). Compared to Beverages, median increases were higher for the MNP for vitamins C (31.2 ± 11.7, *p* < 0.01), E (1.67 ± 0.81, *p* < 0.05), and B12 (0.83 ± 0.27, *p* < 0.01) from baseline to 37 wk; from baseline to 1 mpp, there was a higher median increase in B12 (0.55 ± 0.25, *p* < 0.05) and folate (63.4 ± 24.3, *p* < 0.01); and from baseline to 3 mpp, a higher median increase in iron (2.38 ± 1.06, *p* < 0.05) and folate (94.4 ± 38.1, *p* < 0.05). Conclusions: Intake of micronutrients was higher for MNP and Tablets, likely due to food displacement among Beverage consumers. Although iron bioavailability and absorption inhibitors were not considered for the present analyses, the distribution of Tablets or MNP had several advantages in this context where micronutrient deficiency remains high among pregnant women, but macronutrient intake is generally adequate or even high.

## 1. Introduction

Eating a balanced diet and maintaining a healthy weight during pregnancy is of the utmost importance for optimal maternal and fetal outcomes [1,2,3]. The ability to do so, however, is constrained by economic and social factors in many contexts. The COVID-19 pandemic and continued recovery has emphasized the critical role of social protection programs [1,3]. Nonetheless, increases in economic resources provided by such programs may not adequately address nutrition concerns [4].

To enhance its potential for addressing nutritional deficiencies, the conditional cash transfer program Prospera (initially named Progresa, later Oportunidades, then Prospera, referred to here as CCT-POP) in Mexico distributed a fortified beverage (Beverage) known as Nutrivida to pregnant and lactating women living in poverty from 1997 to 2018 [5,6,7,8,9]. In Mexico, micronutrient deficiency coexists with a high prevalence of overweight and obesity among women, even among women living in poverty [10,11]. The national prevalence of overweight and obesity in non-pregnant women was 72.5% in 2012 and 75.2% in 2018 [12]. At the same time, 33.8% of reproductive age women had a zinc deficiency in 2006 and 25.7% had an iron deficiency in 2018 [13,14,15]. In 2018, the prevalence of anemia among women living in poverty was 34.3% [16].

In the poorest people, inadequate intake of essential micronutrients (folates, vitamin B12, retinol, iron) affects several functional outcomes, including mental and motor development in newborns; nevertheless, supplementation should not replace healthy food consumption [3,5].

A cluster randomized trial involving CCT-POP beneficiary women was designed to assess the differential effect of three supplement groups: a fortified beverage, micronutrient powder (MNP), and micronutrient tablets (Tablets) on various outcomes. All three supplements reduced iron deficiency and there was no differential change in weight gain during pregnancy or weight retention up to 3 months postpartum among the women [5]. At the time, this formed the basis for a recommendation to replace the beverage with tablets (based on outcome, cost, and preference criteria) [6,7]. We revisit these data here to explore the differential effects of the three nutritional supplements on macro- and micronutrient intake of CCT-POP beneficiary pregnant women. The results of this analysis could help understand how women’s diets change with the provision of diverse supplements. This type of information is fundamental to increase the impact potential of social protection programs that include nutrition-specific actions. Our objective was to estimate the differential effects of three nutritional supplements on macro and micronutrient intake of pregnant CCT-POP female beneficiaries.

## 2. Materials and Methods

### 2.1. Study Design

This study is a cluster randomized trial of 723 pregnant women (<25 weeks) beneficiaries of the CCT-POP in four Mexican states (Veracruz, Puebla, Oaxaca, and Tabasco). The study was registered before recruitment began on the Clinical Trial Registry (www.clinicaltrials.gov (accessed on15 July 2020); NCT00531674). More details of the design, study population, intervention, information collection, and primary outcomes were published previously [5]. Briefly, all localities with ≥20,000 habitants, in which at least 70% of the population were beneficiaries of the CCT-POP program, were eligible. We used a block randomized design, considering relevant community level factors (degree of marginalization, population size, and geographic proximity of communities based on national population statistics) using structural equation modeling [5]. The unit of randomization was the community, and the trial was unblinded given supplement presentation differences (Beverage, MNP and Tablets). Communities within blocks were randomly assigned to 1 of 3 supplements (18 communities per supplement) using the “rand” command in Microsoft Excel.

Each supplement, which was produced specifically for this study, provided iron, zinc, folic acid, vitamin C, vitamin E, and vitamin B12 in the same quantity (Table 1).

The supplements contained 100% of the recommended dietary intake for pregnant women, except for vitamin C, which was increased to promote iron absorption. The fortified Beverage also included nutrients that were naturally provided by its ingredients, including 20% of energy and macronutrients (250 kcal/day, 25.3 g/day carbohydrates, and 11.2 g/day lipids) [5,6,7,8,9]. The Beverage supplement was produced for CCT-POP by Liconsa Corporation, (Queretaro, México) and distributed in 264 g bags for a recommended daily dose of 52 g of dry product, which needs to be mixed with a small amount of water prior to consumption. The MNP supplement was purchased from Ped-Med Ltd. (Toronto, Canada) in individual packs with a daily dose of 1 g (to be added to a small portion of food before consumption). The Tablets (1 daily, with 60 Tablets in a pack) were provided as a donation by Zerboni Laboratories in Mexico City, using a premix donated by DMS Nutritional Products (Mexico City). As part of the CCT-POP program, all pregnant and lactating women in the selected communities had been regularly receiving the fortified Beverage and health education messages [5].

### 2.2. Recruitment and Supplement Delivery

#### 2.2.1. Inclusion and Exclusion Criteria

**Figure 1:**
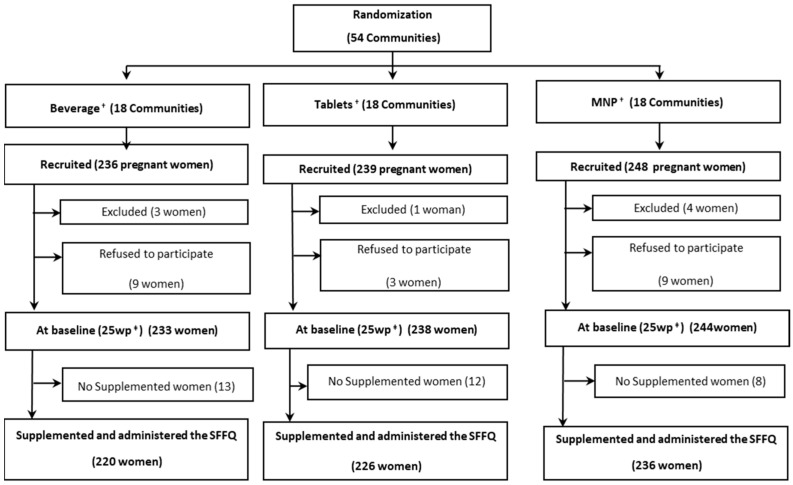
Participant recruitment, exclusion and inclusion at baseline ^†,‡^. ^†^ Beverage: fortified beverage, Tablets: micronutrient tablets, and MNP: micronutrient powder. ^‡^ 25 wk: 25 weeks of pregnancy.

All adult (>18 y) pregnant women (at <25 weeks of gestation, without known pregnancy complications) in the selected communities (18 communities) were identified through beneficiary listings at the corresponding health center and were recruited individually. Exclusion criteria for women included multiple pregnancies and hemoglobin <90 g/L. A sample of 238 women per group was estimated with 80% power and significance level of 5%. The final sample was as follows: Beverage = 220 women, Tablets = 226 women, and MNP = 236 women (Figure 1).

#### 2.2.2. Supplement Delivery

Beverage, MNP, or Tablet supplements were delivered directly to women’s homes six days a week during the first six months of the project; subsequently, staff visits were performed on a weekly basis.

#### 2.2.3. Data Collection

Baseline demographic variables were collected during home visits (age, completed years of education, housing conditions and appliances, parity, and ethnicity) [10]. Weight was measured twice at baseline using an electronic scale (Tanita Corp.) by field staff trained according to international protocols [17]. Height was measured twice at baseline using a portable stadiometer (ShorrBoard^®^) with standard procedures [18]. Ethnicity was recorded if women self-identified as indigenous. Gestational age of women was assessed by recall of last menstrual period using previously validated methods [19]. At each follow-up (scheduled as close as possible to 37 weeks of pregnancy [37 week], and 1 and 3 months postpartum [1 mpp and 3 mpp]), any updates to demographic data, diet, and supplement consumption were noted.

#### 2.2.4. Dietary Intake

Dietary intake was assessed at each follow-up visit by trained health personnel (in Mexican standardized food portion sizes based on the average weight value assigned to each food item) during in-person interviews, using a previously validated (in Mexican women), semi-quantitative, 7-day food frequency questionnaire (SFFQ) [20,21]. The most consumed foods by Mexican women were included and grouped into 15 predefined categories. Women were asked to recall the number of days and the number of times per day they consumed each food item. All data were entered into Epi Info 2000 twice and double checked at the National Institute of Public Health of Mexico (INSP for its acronym in Spanish).

#### 2.2.5. Energy, Nutrient Intake, and Food Group Estimation

Foods were coded and transformed into daily energy and nutrient intake for each evaluation period, using the food composition table developed at the INSP [22]. Energy and nutrient consumption of each supplement was estimated from the week prior to the SFFQ interview date. The total number of doses consumed was obtained by combining partial doses [5]. Daily mean dose consumed was calculated as the total number of doses divided by the number of days reported. For energy and each nutrient, a composite variable was generated using the total nutrient intake from diet plus the nutrient contribution from supplements. Folic acid was converted to dietary folate equivalents (bioavailability = folic acid × 1.7) [23]. Iron bioavailability and iron absorption inhibitors were not considered for the present analyses. Women with incomplete information from FFQ were excluded from the analysis. In total, we analyzed diet information of 200 women who received a beverage, 206 who received tablets, and 192 who received MNP.

Foods were categorized into nine food groups: dairy, fast food, legumes, drinks, snacks/candies/dessert, soups/cream/paste, fruits/vegetables, meat/sausage/egg, and cereals/root crop. For each woman and food group, total energy intake (kcal) was calculated at 37 wk, 1 mpp, and 3 mpp.

#### 2.2.6. Body Mass Index and Socioeconomic Status Index

Body Mass Index (BMI, kg/m^2^) was calculated and categorized according to the World Health Organization (WHO): underweight <18.5, normal 18.5 to 24.9, overweight 25 to 29.9, and obesity >30 [24]. A socioeconomic status (SES) index was constructed (using the full sample) from the primary material of which the dwelling was made (floor, walls, and ceiling), availability of infrastructure services (water source, electricity, and sanitary facilities), and possession of durable goods (e.g., automobile, motorcycle, television, stereo, blender, refrigerator, etc.). The SES index was obtained from the first component of such characteristics and retained 16.3% of the total variation. The index was standardized (zero mean and unit variance) and ranged from −3.6 to 3.5 [25].

### 2.3. Ethics

Written permission was obtained from national and state level health authorities and written informed consent was obtained individually from women who were willing to participate after receiving full details of the study. The study protocol was approved by the Ethics, Biosecurity, and Research Boards of the INSP (CI-213) and was carried out in accordance with the ethical standards outlined in the 1964 Declaration of Helsinki and its later amendments. Recruitment continued from November 2005 to January 2006 and field work was finished in October 2007.

### 2.4. Statistical Analyses

General characteristics and baseline diet intake were compared between study groups using medians and interquartile intervals for quantitative variables and proportions for categorical variables. We compared subjects who were included in the analysis (200 Beverage, 206 Tablets, and 192 MNP) and those not included (only 117 with sociodemographic information) through t-tests for means. Median changes of nutrient consumption from baseline to the other study stages were estimated by median regression with indicator variables of the study groups and design blocks as predictors. For covariate-adjusted analyses, unbalanced characteristics at baseline were identified between study groups and were added to the model predictor. Covariate-adjusted median changes by study group were obtained through predictive margins and pairwise contrasts between study groups were performed through Wald tests. The same model specifications were fitted using changes of nutrients plus supplement consumption as outcome variables. To assess median energy intake by food group and study group, median regressions were fitted for 37 wk, 1 mpp, and 3 mpp, adjusted by unbalanced characteristics at baseline. All standard errors were adjusted for data dependencies at the community level [26]. Statistical significance was reported at *p* < 0.05. Data were analyzed using Stata, Version 15.0 (Stata Co., Santa Monica, SA, USA).

## 3. Results

### 3.1. Baseline Characteristics

Generally, the groups were well balanced at baseline in terms of maternal characteristics (Table 2), with some exceptions. The Tablets group had a higher prevalence of overweight compared to Beverage (42.3% vs. 34.0%), whereas obesity was higher in the Beverage group, compared to the Tablets group (28.9% vs. 19.4%). There were also some differences in the distribution of education level categories and the SES index. These results highlight the importance of adjusting for these variables with multiple regression analyses. Further, women excluded from the analysis tended to have a higher median SES.

### 3.2. Covariate-Adjusted Median Changes of Nutrient Consumption from Diet Alone

Covariate-adjusted median changes in the consumption of carbohydrates, protein, vitamin C, vitamin E, and dietary folate equivalents showed that over 50% of women decreased their dietary intake from 25 wk to 37 wk in all study groups (Table 3). The median change in intake of all other nutrients from 25 wk to 37 wk was negative and statistically significant for the Beverage and Tablets groups. Changes summarized by the median statistic indicated that consumption reductions of zinc, vitamin C, and vitamin B12 were less pronounced in MNP compared to Beverage. For example, the median change of vitamin C was 28.5 mg (±10.3, *p* < 0.01), which was higher in MNP compared to Beverage (−33.9 vs. −62.5).

Median changes from baseline to 1 mpp were generally negative and statistically significant, although the median dietary folate change was higher (less negative) for MNP compared to Beverage. Median changes from baseline to 3 mpp showed a similar pattern, with higher median changes of dietary folate equivalents and iron for MNP compared to the Beverage group, and a higher median change of dietary folate equivalents for Tablets compared to the Beverage group.

### 3.3. Covariate-Adjusted Median Changes of Nutrient Consumption from the Diet Plus Supplements

Median changes of macronutrient intake from dietary and supplement sources tended to be lower in general for MNP and Tablets compared to the Beverage group. With the exception of vitamin C, median changes of micronutrient consumption were all positive and statistically significant in the three study groups, i.e., more than 50% of women increased their consumption of micronutrients from baseline to any other study period (Table 4). These changes were positive and statistically higher for MNP compared to the Beverage group for vitamin C, vitamin E, and vitamin B12 from baseline to 37 wk; for vitamin B12 and dietary folate equivalents from baseline to 1 mpp; and for iron and dietary folate equivalents from baseline to 3 mpp. Regarding macronutrients, the median change from baseline to 37 wk was lower in MNP compared to the Beverage group for protein and lower in Tablets compared to the Beverage group for carbohydrates and protein.

Median changes without covariate adjustments are available in the Supplementary Materials, for nutrient intake from diet and diet plus supplements (Tables S1 and S2).

### 3.4. Adherence to Supplementation

Mean adherence to supplementation during the week prior to the 37 wk SFFQ interview date was 85.6% (95%CI: 80.7, 90.5); for the Beverage group, 89.5% (84.1, 94.9) for the Tablets group, and 92.5% (89.4, 95.7) for MNP. During the week prior to the 1 mpp SFFQ, it was 89.0% (84.0, 94.0) for the Beverage group, 88.6% (83.6, 93.5) for the Tablets group, and 89.3% (84.0, 94.0) for MNP. During the week prior to the 3 mpp SFFQ, it was 78.5% (73.3, 83.6) for the Beverage group, 84.4% (74.4, 94.4) for the Tablets group, and 83.3% (79.1, 87.6) for MNP. There were significant differences in adherence between supplementation groups for MNP vs. the Beverage group only at 37 wk (6.9 ± 2.9, *p* = 0.021).

### 3.5. Food Groups

Covariate-adjusted median energy intake of dairy was lower in the Beverage group compared to MNP (difference ± standard error: 52.2 ± 25.7, *p* = 0.042) at 37 wk. Our results suggest that the median energy intake of fruits and vegetables at 1 mpp was lower in the Beverage group compared to the Tablets (35.6 ± 18.2, *p* = 0.051) and MNP (49.7 ± 26.3, *p* = 0.059) groups, whereas it was higher at 3 mpp in the Tablets group compared to the Beverage (51.1 ± 13.2, *p* < 0.001) and MNP (41.8 ± 16.2, *p* = 0.010) groups (Table S3).

## 4. Discussion

The results of this cluster randomized trial illustrate that, in general, intake of energy and several micronutrients from home foods decreased among pregnant and lactating women consuming a fortified Beverage, compared to women consuming micronutrient-only supplements (MNP and Tablets). Even when considering nutrients from the supplements that were consumed, several differences persisted, leading to higher intake of several micronutrients among MNP consumers. Median changes of macronutrient intake from dietary plus supplement sources tended to be lower in general for MNP and Tablets compared to Beverage. These findings suggest that in the Beverage group, regularly consumed foods at home (dairy, fruits, and vegetables) were replaced by the Beverage. It is likely that the displaced foods were poorer sources of protein than the Beverage (given its milk base). Generally, however, protein consumption is not limited in this population. MNP showed a higher increase in micronutrient dietary intake alone, perhaps due to slightly higher consumption, although these differences were significant mainly in the first period [23,27].

Supplementation adherence in this study was very high, above 80% in all groups and study periods. This is likely due to the close follow-up and motivational approach taken to promote the supplement. In the CCT-POP program context, where promotion for consumption of the product was minimal, a cohort study found that only 25% of lactating women reported consuming the Beverage [28]. According to the WHO [29], maternal adherence to MNP is lower than adherence to iron and folic acid supplementation (relative risk = 0.76, 95%CI:0.66, 0.87; *n* = 405). According to Suchdev et al. [30], adherence to routine supplementation regimens during pregnancy is only about 50% due to constipation and nausea associated with supplemental iron. Our results suggest that with encouragement and motivational prompts, higher adherence is feasible. This is critical to achieve intended impacts.

Interestingly, in the current study, adherence to supplementation did not decrease even after daily visits were discontinued [5,8]. It is possible that the weekly visits and memory prompts provided as part of the study provided the needed reminders and motivation for women to continue supplementation [31]. Other studies have similarly reported the potential positive role of family members and peers to support and encourage adherence to prenatal micronutrient supplementation [32,33]. The women from our study also received prenatal care and this may have improved their knowledge of anemia and enhanced adherence, as has been suggested elsewhere [5,28,33,34,35,36,37,38].

According to Neufeld et al. [5], no differences were found in the change in weight from baseline to 37 wk, 1 mpp, or 3 mpp between these three groups, or in weight retention up to 3 months postpartum among the women. Nevertheless, given the high prevalence of overweight and obesity in the current Mexican context, micronutrient-only supplements may be more suitable for population level interventions [39]. This situation may be similar in other contexts where overweight and obesity prevalence are high. However, the finding that the Beverage provided additional protein could be important if intake is insufficient in the diet; this was not the case in our study population [38,39]. Importantly, supplementation programs should not be carried out at the cost of ensuring effective strategies are in place to increase availability and affordability of natural foods that are rich in essential nutrients in communities with high rates of poverty [38,39].

Our study has several limitations. Individual and group nutrient intakes obtained from the SFFQ instrument did not reproduce adequately true usual intake levels, as this instrument is limited to a determined number of food items. However, analyses of relative quantities (e.g., differences across time or across groups) could be less prone to estimation bias. A better estimation of intake levels would require repeated 24 h recalls, which was considered infeasible as part of this study due to field logistics [21,40]. Another limitation concerns diet consumption reporting when participants are aware of being enrolled in the study. It is possible that they may have emphasized consumption of healthy foods and under-reported consumption of non-healthy foods, a limitation that may be similar regardless of the dietary assessment method used [21,40]. Additionally, iron bioavailability and iron absorption inhibitors were not considered for the present analyses, and neither was seasonal variability, which probably influenced changes in dietary consumption. For logistical reasons, the study was implemented in small and medium-sized urban areas, and there may be some limitations to extrapolations of our results to all rural areas of Mexico. That said, previous research suggests that the prevalence of overweight and obesity varies little in the CCT-POP population from urban to rural regions, and the prevalence of micronutrient deficiency may even be higher in rural regions [13,14,15].

The study also has several strengths, particularly the longitudinal data on a relatively large group of women that permitted the assessment of individual changes between baseline and several follow-up points. This allowed us to control for both observed and unobserved factors with time invariant effects on the outcomes. On the other hand, potential reporting and instrument biases were not related to study groups by design, allowing us to compare differences between study groups. As an additional strength, continuous and rigorous monitoring of supplement consumption was performed by highly trained field workers in our study. Finally, the information reported here was used immediately to adapt the program in Mexico, considering the totality of evidence related to the supplements [5]. That said, the implications and conclusions related to the importance of exploring the potential of nutritional products as part of programs remains highly relevant.

In many parts of the world, the provision of nutritional supplements forms a critical component of integrated care for pregnant and lactating women [3]. The choice of such supplements should be based on the specific nutritional profile and needs of the population [41,42]. In Mexico, the high prevalence of overweight and obesity combined with persistent micronutrient deficiencies prompted us to study the potential for switching from a fortified Beverage to one of two alternative micronutrient-only supplements. The results reported here support our previous findings that the fortified Beverage can be replaced with a micronutrient-only supplement because there was no difference between groups regarding changes in hemoglobin concentration or prevalence of anemia from baseline to 37 weeks of gestation or 1 or 3 mpp. In addition, the total cost of production and distribution for the Beverage was USD 0.173/dose, for Tablets, USD 0.056/dose, and MNP, USD 0.023/dose [5]. According to Neufeld et al. [5], considering the program recommendations of supplementation for 18 months, a switch from the Beverage to MNP would result in a saving per beneficiary of USD 82.2, translating into yearly savings of USD 29 million (given that 362,000 women were receiving supplements at the time of the study). This recommendation was taken up by the program and the modification from the fortified Beverage to Tablets was made shortly after completion of this trial. The CCT-POP program finished in 2018 and distribution of the supplements and related promotion was discontinued. Unfortunately, since then, anemia has increased (34.3%), becoming a public health problem among women once again [5,16].

In this post-COVID area, the prevention and management of micronutrient deficiency urgently needs to be elevated as part of the public health agenda. Deficiencies can exacerbate the severity and consequences of several infections [42,43,44]. Indeed, some studies have proposed nutritional supplements (macronutrients and micronutrients) as a possible adjuvant treatment in viral infections, ameliorating the inflammatory and oxidative stress associated with the disease and some direct antiviral effects [43,44].

## Figures and Tables

**Table 1:** Nutritional content of supplements delivered to mothers.

	Supplements (Dose)		
	Beverage ^†, ‡^	Tablets ^†^	MNP ^†^
Energy (kcal)	250	-	-
Protein (g)	12	-	-
Carbohydrates (g)	25.3	-	-
Lipid (g)	11.2	-	-
Sodium (mg)	81.2	-	-
Iron (mg) ^Φ^	15	15	15
Zinc gluconate (mg)	15	15	15
Iodine (µg)	100	100	100
Folic acid (µg)	400	400	400
Ascorbic acid (mg) ^§^	100	100	100
Potassium Iodine (µg)	100	100	100
Vitamin E (Acetate; mg ET)	10	10	10
Vitamin B12 (Cyanocobalamin; µg)	2.6	2.6	2.6

^†^ Beverage: fortified beverage, Tablets: micronutrient tablets, and MNP: micronutrient powder. ^‡^ Beverage supply 20% of energy; the three supplements supply 100% of micronutrients daily. ^Φ^ Ferrous gluconate used in Tablets and Beverage; ferrous fumarate in MNP. Same form used in all three supplements for other nutrients. ^§^ Ascorbic acid or Vitamin C.

**Table 2:** Characteristics of the women at baseline ^†^ by evaluation study groups.

	Beverage ^‡^	Tablets ^‡^	MNP ^‡^	Excluded
	*n* = 200	*n* = 206	*n* = 192	*n* = 117 ^£^
Age	27.7 [22.2, 32.9]	26.6 [23.4, 31.2]	26.9 [22.8, 31.4]	26.0 [21.4, 30.8]
Weight, kg	59.0 [52.7, 68.9]	58.4 [52.7, 65.4]	60.5 [53.1, 66.7]	60.0 [53.0, 67.3]
Height, cm	149.4 [146.0, 152.9]	150.0 [145.7, 154.6]	150.0 [147.4, 153.9]	-
BMI, kg/m^2^	26.6 [23.4, 30.9]	26.3 [23.4, 28.9]	26.4 [23.2, 29.7]	-
Overweight, %	34.0	42.3	39.3	39.5
Obesity, %	28.9	19.4	23.0	19.8
SES Index, SD ^¥^	0.1 [−0.5, 0.7]	0.0 [−0.6, 0.6]	−0.1 [−0.8, 0.5]	0.2 [−0.4, 0.8]
Primiparous, %	18.5	14.1	12.5	19.7
Indigenous, % ^ψ^	7.0	8.8	6.8	7.7
Educational level ^Φ^				
None, %	35.0	25.4	35.9	25.6
Elementary, %	27.0	31.7	32.3	34.2
Middle or higher, %	38.0	42.9	31.8	40.2
Diet intake alone				
Energy, kcal	1890 [1426, 2569]	1743 [1141, 2445]	1826 [1338, 2606]	1836 [1340, 2847]
Carbohydrates, g	258.0 [195.5, 351.3]	234.6 [156.3, 336.8]	241.5 [183.7, 351.5]	249.7 [183.3, 342.1]
Protein, g	61.9 [42.4, 83.3]	55.8 [35.7, 78.9]	61.5 [44.7, 83.8]	62.0 [42.1, 88.1]
Lipids, g	74.2 [46.1, 104.0]	65.7 [39.0, 94.0]	71.0 [44.7, 127.0]	70.9 [46.0, 114.9]
Iron, mg	12.4 [8.6, 16.9]	10.9 [7.7, 15.8]	11.7 [8.6, 17.4]	11.8 [8.0, 17.1]
Zinc, mg	8.9 [6.0, 12.0]	8.2 [5.3, 11.3]	8.6 [6.1, 11.8]	8.5 [5.9, 12.4]
Vitamin C, mg	168.6 [108.1, 318.3]	161.6 [94.1, 289.5]	196.7 [111.7, 295.7]	181.5 [95.7, 293.4]
Vitamin E, mg	10.3 [5.1, 16.1]	9.1 [4.4, 14.7]	9.7 [4.7, 19.6]	8.6 [4.7, 15.5]
Vitamin B12, mcg	3.6 [2.4, 5.5]	3.2 [1.7, 4.9]	3.8 [2.3, 5.3]	3.5 [2.3, 6.0]
DFE, mcg ^§^	416.3 [302.2, 648.9]	403.4 [241.8, 573.7]	458.3 [293.5, 715.4]	428.2 [265.7, 651.2]

Estimates are medians [P25, P75] or percentages. ^†^ 25 wk: 25 weeks of pregnancy. ^‡^ Beverage: fortified beverage, Tablets: micronutrient tablets, and MNP: micronutrient powder. ^Φ^ Education level was assessed by the number of years of formal schooling completed and was stratified into four categories: <1 = none, 1–6 years = elementary, >7 years = middle or higher. ^¥^ SD: standard deviation. ^ψ^ Self-identified as indigenous peoples. ^§^ DFE = dietary folate equivalents (bioavailability = folic acid × 1.7). ^£^ Women who were excluded, did not participate, or had incomplete information from FFQ, but information of sociodemographic characteristics was still possible to obtain.

**Table 3:** Covariate-adjusted median changes ^†^ of nutrient intake from diet alone, by evaluation study groups ^‡^ and period ^Φ^.

	Beverage	Tablets	MNP	Tablets vs. Beverage	MNP vs. Beverage	MNP vs. Tablets
Changes from baseline to 37 wk						
	*n* = 152	*n* = 168	*n* = 157			
Energy, kcal	−239.9 *** ± 65.6	−260.9 ** ± 89.8	−77.7 ± 67.2	−20.9 ± 118.4	162.2 ± 97.7	183.2 ± 115.8
Carbohydrates, g	−41.5 *** ± 8.5	−43.7 *** ± 8.6	−25.8 *** ± 7.8	−2.2 ± 12.2	15.7 ± 11.3	17.9 ± 12.0
Protein, g	−7.71 *** ± 1.85	−9.16 *** ± 2.44	−4.91 * ± 2.08	−1.45 ± 3.11	2.80 ± 2.74	4.25 ± 3.43
Lipids, g	−14.53 *** ± 3.09	−12.43 ** ± 4.25	−7.05 ± 4.02	2.10 ± 6.10	7.48 ± 5.14	5.38 ± 5.82
Iron, mg	−1.65 *** ± 0.43	−1.88 ** ± 0.66	−0.64 ± 0.43	−0.23 ± 0.84	1.00 ± 0.56	1.24 ± 0.89
Zinc, mg	−1.31 *** ± 0.30	−1.41 *** ± 0.39	−0.30 ± 0.27	−0.10 ± 0.52	1.01 ** ± 0.39	1.10 * ± 0.52
Vitamin C, mg	−62.5 *** ± 8.8	−47.2 *** ± 8.1	−33.9 *** ± 7.3	15.3 ± 12.8	28.5 ** ± 10.3	13.3 ± 12.0
Vitamin E, mg	−3.48 *** ± 0.57	−3.30 *** ± 0.63	−2.29 *** ± 0.55	0.18 ± 0.89	1.19 ± 0.74	1.01 ± 0.88
Vitamin B12, mcg	−0.88 *** ± 0.19	−0.56 ** ± 0.20	−0.10 ± 0.18	0.32 ± 0.28	0.78 ** ± 0.28	0.46 ± 0.30
DFE, mcg ^§^	−80.0 ** ± 24.7	−98.0 ** ± 31.1	−61.9 ** ± 21.6	−18.0 ± 45.0	18.0 ± 28.5	36.1 ± 41.7
Changes from baseline to 1 mpp						
	*n* = 188	*n* = 197	*n* = 178			
Energy, kcal	−319.5 *** ± 67.5	−176.8 * ± 81.0	−189.0 * ± 75.4	142.7 ± 102.7	130.5 ± 87.0	−12.2 ± 110.2
Carbohydrates, g	−40.6 *** ± 8.3	−34.1 ** ± 12.2	−37.1 *** ± 7.2	6.4 ± 14.3	3.5 ± 10.0	−2.9 ± 12.5
Protein, g	−5.33 ** ± 1.76	−1.25 ± 2.60	−1.80 ± 2.26	4.08 ± 3.22	3.53 ± 2.68	−0.55 ± 3.64
Lipids, g	−20.49 *** ± 2.73	−14.37 ** ± 4.61	−18.37 *** ± 3.86	6.12 ± 5.50	2.12 ± 4.60	−4.00 ± 6.03
Iron, mg	−2.56 *** ± 0.56	−1.69 *** ± 0.48	−1.46 ** ± 0.48	0.87 ± 0.76	1.10 ± 0.65	0.23 ± 0.74
Zinc, mg	−1.29 *** ± 0.29	−0.71 ± 0.38	−0.65 ± 0.40	0.58 ± 0.48	0.64 ± 0.50	0.06 ± 0.63
Vitamin C, mg	−113.9 *** ± 7.7	−118.2 *** ± 6.8	−114.7 *** ± 6.1	−4.3 ± 11.2	−0.8 ± 9.2	3.5 ± 9.5
Vitamin E, mg	−6.35 *** ± 0.50	−5.60 *** ± 0.56	−5.55 *** ± 0.54	0.75 ± 0.75	0.80 ± 0.62	0.04 ± 0.69
Vitamin B12, mcg	−0.65 *** ± 0.17	−0.46 ± 0.25	−0.22 ± 0.19	0.19 ± 0.26	0.43 ± 0.24	0.24 ± 0.32
DFE, mcg ^§^	−181.5 *** ± 18.1	−156.6 *** ± 16.9	−131.3 *** ± 17.2	24.9 ± 22.7	50.2 * ± 23.9	25.3 ± 25.5
Changes from baseline to 3 mpp						
	*n* = 187	*n* = 198	*n* = 169			
Energy, kcal	−334.2 *** ± 70.7	−223.0 * ± 89.5	−212.1 * ± 90.9	111.2 ± 116.3	122.1 ± 125.7	10.9 ± 139.1
Carbohydrates, g	−56.2 *** ± 10.0	−49.4 *** ± 10.5	−38.5 ** ± 14.9	6.9 ± 12.7	17.8 ± 19.0	10.9 ± 19.9
Protein, g	−9.10 *** ± 2.28	−6.51 ** ± 2.40	−4.25 ± 3.44	2.59 ± 3.49	4.85 ± 4.22	2.26 ± 4.29
Lipids, g	−14.92 ** ± 4.54	−5.42 ± 5.11	−9.38 * ± 3.86	9.50 ± 6.67	5.54 ± 5.45	−3.96 ± 6.05
Iron, mg	−2.45 *** ± 0.48	−1.74 *** ± 0.48	−0.72 ± 0.62	0.71 ± 0.63	1.73 * ± 0.82	1.02 ± 0.80
Zinc, mg	−1.22 ** ± 0.39	−1.16 ** ± 0.36	−0.36 ± 0.49	0.06 ± 0.52	0.86 ± 0.66	0.80 ± 0.63
Vitamin C, mg	−108.7 *** ± 7.6	−94.1 *** ± 5.5	−96.1 *** ± 8.8	14.6 ± 10.2	12.6 ± 11.8	−2.1 ± 10.5
Vitamin E, mg	−4.53 *** ± 0.68	−3.90 *** ± 1.05	−4.25 *** ± 0.69	0.63 ± 1.21	0.28 ± 0.97	−0.35 ± 1.28
Vitamin B12, mcg	−0.59 * ± 0.23	−0.49 * ± 0.24	−0.32 ± 0.23	0.10 ± 0.36	0.27 ± 0.30	0.17 ± 0.33
DFE, mcg ^§^	−163.6 *** ± 20.1	−108.0 *** ± 16.3	−83.3 *** ± 24.7	55.5 * ± 27.1	80.3 ** ± 30.1	24.8 ± 30.8

Estimates are median changes or differences of median changes between study groups ± standard error. * *p* < 0.05, ** *p* < 0.01, *** *p* < 0.001. ^†^ Obtained from quantile regression and adjusted for baseline intake, SES index, education level, and BMI. ^‡^ Beverage: fortified beverage, Tablets: micronutrient tablets, and MNP: micronutrient powder. ^Φ^ 25 wk: 25 weeks of pregnancy (baseline), 37 wk: 37 weeks of pregnancy, 1 mpp: 1 month postpartum, 3 mpp: 3 months postpartum. ^§^ DFE = dietary folate equivalents (bioavailability= folic acid × 1.7).

**Table 4:** Covariate-adjusted median changes ^†^ of nutrient intake from diet plus supplements, by evaluation study groups ^‡^ and period ^Φ^.

	Beverage	Tablets	MNP	Tablets vs. Beverage	MNP vs. Beverage	MNP vs. Tablets
Changes from baseline to 37 wk						
	*n* = 152	*n* = 168	*n* = 157			
Energy, kcal	−25.0 ± 83.2	−264.6 ** ± 94.3	−89.2 ± 70.0	−239.6 ± 141.5	−64.2 ± 109.7	175.4 ± 120.3
Carbohydrates, g	−16.1 ± 8.5	−44.4 *** ± 10.0	−23.5 ** ± 8.4	−28.4 * ± 13.2	−7.4 ± 12.0	20.9 ± 14.0
Protein, g	2.60 ± 1.90	−8.94 *** ± 2.38	−5.20 * ± 2.20	−11.54 *** ± 3.00	−7.80 ** ± 2.90	3.74 ± 3.54
Lipids, g	−4.22 ± 3.39	−12.83 ** ± 4.15	−6.96 ± 3.93	−8.60 ± 6.33	−2.73 ± 5.18	5.87 ± 5.60
Iron, mg	11.94 *** ± 0.47	12.01 *** ± 0.53	12.69 *** ± 0.32	0.08 ± 0.71	0.75 ± 0.54	0.68 ± 0.69
Zinc, mg	13.00 *** ± 0.44	12.74 *** ± 0.42	13.61 *** ± 0.33	−0.26 ± 0.64	0.61 ± 0.50	0.86 ± 0.63
Vitamin C, mg	25.2 *** ± 7.6	40.7 *** ± 9.5	56.3 *** ± 8.8	15.5 ± 13.5	31.2 ** ± 11.7	15.6 ± 14.7
Vitamin E, mg	5.39 *** ± 0.64	5.63 *** ± 0.76	7.07 *** ± 0.70	0.24 ± 1.06	1.67 * ± 0.81	1.44 ± 1.13
Vitamin B12, mcg	1.48 *** ± 0.19	1.66 *** ± 0.22	2.31 *** ± 0.17	0.18 ± 0.31	0.83 ** ± 0.27	0.65 * ± 0.31
DFE, mcg ^§^	546.8 *** ± 25.7	540.5 *** ± 36.2	587.6 *** ± 21.2	−6.3 ± 49.5	40.8 ± 30.4	47.1 ± 47.3
Changes from baseline to 1 mpp						
	*n* = 188	*n* = 197	*n* = 178			
Energy, kcal	−103.3 ± 63.4	−200.0 * ± 86.9	−175.4 * ± 75.0	−96.8 ± 103.2	−72.2 ± 90.0	24.6 ± 116.5
Carbohydrates, g	−22.8 ** ± 8.8	−31.7 ** ± 11.4	−35.8 *** ± 7.5	−8.9 ± 13.9	−13.0 ± 10.7	−4.1 ± 13.0
Protein, g	5.50 ** ± 2.12	−0.82 ± 2.45	−1.84 ± 2.23	−6.32 ± 3.31	−7.34 * ± 2.90	−1.02 ± 3.42
Lipids, g	−9.76 *** ± 2.61	−14.43 ** ± 4.51	−18.12 *** ± 3.72	−4.67 ± 5.35	−8.36 ± 4.38	−3.69 ± 5.83
Iron, mg	11.35 *** ± 0.57	11.64 *** ± 0.58	12.41 *** ± 0.49	0.28 ± 0.87	1.05 ± 0.73	0.77 ± 0.79
Zinc, mg	12.86 *** ± 0.46	13.21 *** ± 0.42	13.63 *** ± 0.36	0.35 ± 0.59	0.77 ± 0.62	0.42 ± 0.63
Vitamin C, mg	−19.1 * ± 7.7	−26.2 *** ± 7.2	−18.9 ** ± 5.8	−7.2 ± 9.9	0.1 ± 9.0	7.3 ± 9.3
Vitamin E, mg	3.21 *** ± 0.53	3.67 *** ± 0.59	3.62 *** ± 0.50	0.46 ± 0.78	0.41 ± 0.59	−0.05 ± 0.70
Vitamin B12, mcg	1.72 *** ± 0.19	1.77 *** ± 0.21	2.27 *** ± 0.18	0.04 ± 0.26	0.55 * ± 0.25	0.50 ± 0.28
DFE, mcg ^§^	444.1 *** ± 22.5	490.7 *** ± 24.8	507.5 *** ± 14.1	46.7 ± 33.2	63.4 ** ± 24.3	16.8 ± 28.4
Changes from baseline to 3 mpp						
	*n* = 187	*n* = 198	*n* = 169			
Energy, kcal	−142.6 ± 80.0	−235.0 * ± 103.9	−207.3 * ± 95.5	−92.3 ± 136.3	−64.6 ± 138.2	27.7 ± 156.1
Carbohydrates, g	−34.0 *** ± 9.8	−48.5 *** ± 10.6	−39.4 * ± 16.2	−14.5 ± 13.0	−5.3 ± 20.5	9.2 ± 20.7
Protein, g	0.08 ± 2.43	−6.46 * ± 2.73	−3.55 ± 3.43	−6.54 ± 3.88	−3.63 ± 4.44	2.91 ± 4.55
Lipids, g	−5.72 ± 4.42	−5.89 ± 4.52	−9.27 * ± 4.19	−0.17 ± 6.34	−3.55 ± 5.76	−3.37 ± 6.04
Iron, mg	10.13 *** ± 0.70	10.93 *** ± 0.77	12.50 *** ± 0.84	0.81 ± 1.08	2.38 * ± 1.06	1.57 ± 1.19
Zinc, mg	11.69 *** ± 0.45	12.26 *** ± 0.62	13.11 *** ± 0.56	0.57 ± 0.84	1.43 ± 0.73	0.85 ± 0.91
Vitamin C, mg	−24.3 *** ± 6.4	−13.4 ** ± 5.1	−8.3 ± 9.5	10.9 ± 8.3	16.0 ± 11.5	5.1 ± 11.0
Vitamin E, mg	3.67 *** ± 0.94	4.77 *** ± 0.84	4.01 *** ± 0.74	1.10 ± 1.15	0.34 ± 0.91	−0.76 ± 1.02
Vitamin B12, mcg	1.48 *** ± 0.22	1.67 *** ± 0.23	1.79 *** ± 0.27	0.19 ± 0.33	0.31 ± 0.37	0.12 ± 0.38
DFE, mcg ^§^	418.3 *** ± 28.9	481.2 *** ± 35.8	512.7 *** ± 28.5	62.9 ± 48.2	94.4 * ± 38.1	31.5 ± 49.0

Estimates are median changes or differences of median changes between study groups ± standard error. * *p* < 0.05, ** *p* < 0.01, *** *p* < 0.001. ^†^ Obtained from quantile regression and adjusted for baseline intake, SES index, education level, and BMI. ^‡^ Beverage: fortified beverage, Tablets: micronutrient tablets, and MNP: micronutrient powder. ^Φ^ 25 wk: 25 weeks of pregnancy (baseline), 37 wk: 37 weeks of pregnancy, 1 mpp: 1 month postpartum, 3 mpp: 3 months postpartum. ^§^ DFE = dietary folate equivalents (bioavailability= folic acid × 1.7).

## Data Availability

Not applicable.

## Supplementary Materials

The following supporting information can be downloaded at: https://www.mdpi.com/article/10.3390/nu14153003/s1, Table S1: Median changes† of nutrient intake from diet alone, by evaluation study groups ^‡^ and period ^Φ^. Table S2: Median changes† of nutrient intake from diet plus supplements, by evaluation study groups ^‡^ and period ^Φ^. Table S3: Covariate-adjusted median† energy intake of food groups from diet alone, by evaluation study groups ^‡^ and period ^Φ^.
