# Supplementary material for: Differential Effects of Three Nutritional Supplements on the Nutrient Intake of Pregnant Women Enrolled in a Conditional Cash Transfer Program in Mexico: A Cluster Randomized Trial
Source: Nutrients. 2022 Jul 22;14(15):3003. doi: 10.3390/nu14153003 (PMC9332738; doi:10.3390/nu14153003)
Supplement: Supplementary file 1 [file nutrients-14-03003-s001.zip › nutrients-1778456-supplementary.pdf]

Table S1. Median changes† of nutrient intake from diet alone, by evaluation study groups ‡ and period Φ

	Beverage	Tablets	MNP	Tablets vs Beverage	MNP vs Beverage	MNP vs Tablets
**Changes from baseline to 37wk**						
	n=152	n=168	n=157			
Energy, kcal	-194.4* ± 77.3	11.8 ± 61.4	31.8 ± 83.8	206.2* ± 97.9	226.2 ± 121.3	20.1 ± 102.9
Carbohydrates, g	-18.2 ± 10.5	-1.5 ± 10.6	-14.3 ± 9.0	16.7 ± 15.0	3.8 ± 14.8	-12.9 ± 14.5
Protein, g	-4.83* ± 2.20	-0.05 ± 2.09	0.17 ± 2.31	4.78 ± 2.70	4.99 ± 3.20	0.21 ± 3.12
Lipids, g	-10.02*** ± 2.75	0.12 ± 3.42	1.86 ± 4.22	10.13* ± 4.79	11.88* ± 5.53	1.74 ± 5.20
Iron, mg	-1.21** ± 0.44	-0.38 ± 0.38	-0.51 ± 0.53	0.83 ± 0.56	0.70 ± 0.75	-0.13 ± 0.65
Zinc, mg	-0.80* ± 0.37	0.09 ± 0.29	0.03 ± 0.32	0.90 ± 0.48	0.83 ± 0.49	-0.07 ± 0.44
Vitamin C, mg	-15.2 ± 12.9	-5.7 ± 8.2	-12.0 ± 10.3	9.6 ± 16.3	3.3 ± 15.2	-6.3 ± 11.6
Vitamin E, mg	-0.21 ± 0.61	-0.01 ± 0.54	-0.10 ± 0.62	0.21 ± 0.87	0.11 ± 0.88	-0.09 ± 0.84
Vitamin B12, mcg	-0.19 ± 0.16	0.09 ± 0.21	0.46* ± 0.19	0.28 ± 0.29	0.65* ± 0.25	0.37 ± 0.29
DFE, mcg §	-45.1* ± 21.4	-8.2 ± 23.0	-9.4 ± 17.5	36.9 ± 32.0	35.7 ± 29.5	-1.3 ± 31.5
**Changes from baseline to 1mpp**						
	n=188	n=197	n=178			
Energy, kcal	-272.8*** ± 51.5	-17.5 ± 58.1	-91.9 ± 66.4	255.3*** ± 75.4	180.9* ± 88.8	-74.4 ± 94.5
Carbohydrates, g	-36.6*** ± 8.5	-12.7 ± 8.1	-23.1** ± 7.8	23.9* ± 12.1	13.5 ± 12.2	-10.4 ± 11.7
Protein, g	-2.95 ± 1.58	3.31 ± 2.32	2.56 ± 2.38	6.27* ± 2.51	5.51 ± 2.89	-0.76 ± 3.37
Lipids, g	-11.95*** ± 2.93	-2.21 ± 2.73	-5.26 ± 3.21	9.74* ± 4.35	6.69 ± 4.06	-3.05 ± 4.42
Iron, mg	-1.48*** ± 0.29	-0.40 ± 0.26	-0.60 ± 0.31	1.08** ± 0.38	0.88* ± 0.42	-0.20 ± 0.43
Zinc, mg	-0.75** ± 0.26	0.03 ± 0.27	0.29 ± 0.33	0.78* ± 0.37	1.04* ± 0.43	0.26 ± 0.45
Vitamin C, mg	-80.4*** ± 12.1	-77.0*** ± 11.8	-72.9*** ± 12.9	3.3 ± 18.7	7.5 ± 19.5	4.2 ± 14.0
Vitamin E, mg	-3.54*** ± 0.65	-1.15* ± 0.47	-2.42** ± 0.77	2.39** ± 0.79	1.12 ± 0.90	-1.27 ± 0.87
Vitamin B12, mcg	-0.41* ± 0.16	0.14 ± 0.16	0.00 ± 0.23	0.54* ± 0.23	0.41 ± 0.25	-0.14 ± 0.30
DFE, mcg §	-105.0*** ± 17.5	-58.3*** ± 16.5	-79.3** ± 25.7	46.7* ± 23.0	25.8 ± 32.3	-21.0 ± 29.4
**Changes from baseline to 3mpp**						
	n=187	n=198	n=169			
Energy, kcal	-195.7*** ± 57.3	-52.4 ± 58.5	-132.7 ± 78.2	143.3 ± 90.6	62.9 ± 98.7	-80.4 ± 92.2
Carbohydrates, g	-43.0*** ± 11.4	-22.1* ± 9.7	-23.9* ± 11.7	20.9 ± 13.0	19.1 ± 17.6	-1.8 ± 14.4

Protein, g	-5.10* ± 2.38	-4.11 ± 2.49	1.19 ± 3.12	0.99 ± 3.57	6.29 ± 4.20	5.31 ± 3.90
Lipids, g	-7.31* ± 3.44	1.96 ± 3.36	1.43 ± 4.60	9.27 ± 5.08	8.74 ± 6.11	-0.53 ± 5.95
Iron, mg	-1.52* ± 0.73	-0.74 ± 0.69	-0.38 ± 0.79	0.78 ± 0.76	1.14 ± 0.90	0.36 ± 0.64
Zinc, mg	-0.85* ± 0.34	-0.44 ± 0.36	-0.13 ± 0.39	0.41 ± 0.54	0.73 ± 0.51	0.32 ± 0.52
Vitamin C, mg	-74.1*** ± 7.6	-50.2*** ± 8.6	-53.4*** ± 12.3	24.0 ± 12.6	20.7 ± 14.9	-3.2 ± 14.5
Vitamin E, mg	-2.05** ± 0.78	-0.28 ± 0.55	-0.92 ± 0.93	1.77* ± 0.90	1.12 ± 1.28	-0.65 ± 1.20
Vitamin B12, mcg	-0.27 ± 0.23	-0.15 ± 0.18	0.10 ± 0.26	0.12 ± 0.31	0.37 ± 0.35	0.25 ± 0.33
DFE, mcg §	-97.0*** ± 26.3	-32.4 ± 19.6	-30.3 ± 23.9	64.6 ± 34.8	66.7 ± 37.2	2.1 ± 32.0

† Estimates are median changes or differences of median changes between study groups ± standard error

*p<0.05,**p<0.01,***p<0.001

‡ Beverage: Fortified beverage, Tablets: Micronutrient tablets and MNP: Micronutrient powder

Φ 25wp: 25 weeks of pregnant (baseline), 37wp: 37 weeks of pregnant, 1mpp: 1 month post-partum, 3 mpp: 3 months post-partum

§ DFE=Dietary folate equivalents (bioavailability= folic acid x 1.7)

Table S2. Median changes† of nutrient intake from diet plus supplements, by evaluation study groups ‡ and period Φ

	Beverage	Tablets	MNP	Tablets vs Beverage	MNP vs Beverage	MNP vs Tablets
**Changes from baseline to 37wk**						
	n=152	n=168	n=157			
Energy, kcal	36.0 ± 83.8	13.6 ± 68.0	33.7 ± 85.1	-22.4 ± 108.9	-2.4 ± 124.6	20.1 ± 108.1
Carbohydrates, g	5.4 ± 10.5	-3.2 ± 11.3	-16.2 ± 10.1	-8.6 ± 15.7	-21.6 ± 15.2	-13.0 ± 16.0
Protein, g	5.49** ± 2.12	0.63 ± 1.96	-0.27 ± 2.44	-4.86 ± 2.67	-5.77 ± 3.29	-0.90 ± 3.18
Lipids, g	0.76 ± 2.96	-0.13 ± 3.47	1.61 ± 4.31	-0.90 ± 5.03	0.85 ± 5.76	1.74 ± 5.29
Iron, mg	11.62*** ± 0.78	14.08*** ± 0.55	13.35*** ± 0.59	2.46* ± 0.97	1.73 ± 1.07	-0.73 ± 0.88
Zinc, mg	12.58*** ± 0.74	14.49*** ± 0.41	13.97*** ± 0.48	1.91* ± 0.89	1.39 ± 0.83	-0.53 ± 0.67
Vitamin C, mg	69.4*** ± 14.5	79.2*** ± 9.8	83.8*** ± 10.8	9.8 ± 18.1	14.3 ± 16.1	4.5 ± 14.0
Vitamin E, mg	8.67*** ± 0.80	9.32*** ± 0.70	8.87*** ± 0.72	0.64 ± 1.14	0.19 ± 1.14	-0.45 ± 1.01
Vitamin B12, mcg	2.13*** ± 0.19	2.41*** ± 0.20	2.84*** ± 0.22	0.28 ± 0.30	0.70* ± 0.30	0.43 ± 0.32
DFE, mcg §	542.8*** ± 34.0	640.0*** ± 35.5	618.0*** ± 19.3	97.2 ± 54.4	75.3 ± 40.2	-21.9 ± 44.8
**Changes from baseline to 1mpp**						
	n=188	n=197	n=178			
Energy, kcal	-51.5 ± 46.9	-23.0 ± 59.3	-97.4 ± 64.3	28.5 ± 75.8	-45.9 ± 83.2	-74.4 ± 94.1
Carbohydrates, g	-16.0 ± 8.7	-9.1 ± 9.8	-20.7** ± 7.2	6.9 ± 13.5	-4.8 ± 11.9	-11.6 ± 12.3
Protein, g	7.15*** ± 1.51	3.90 ± 2.26	2.10 ± 2.64	-3.24 ± 2.68	-5.05 ± 3.30	-1.81 ± 3.59
Lipids, g	-3.55 ± 2.85	-2.22 ± 3.14	-6.13 ± 3.82	1.32 ± 4.59	-2.59 ± 4.44	-3.91 ± 5.17
Iron, mg	12.23*** ± 0.46	13.02*** ± 0.53	13.18*** ± 0.47	0.78 ± 0.69	0.95 ± 0.68	0.16 ± 0.79
Zinc, mg	12.93*** ± 0.43	13.62*** ± 0.55	14.46*** ± 0.49	0.68 ± 0.63	1.52* ± 0.66	0.84 ± 0.79
Vitamin C, mg	10.3 ± 12.7	13.6 ± 11.9	14.9 ± 14.5	3.3 ± 19.8	4.6 ± 22.2	1.3 ± 15.2
Vitamin E, mg	5.29*** ± 0.62	7.31*** ± 0.58	5.93*** ± 0.64	2.02* ± 0.93	0.64 ± 0.87	-1.38 ± 0.91
Vitamin B12, mcg	1.83*** ± 0.21	2.36*** ± 0.19	2.49*** ± 0.22	0.53 ± 0.29	0.66* ± 0.28	0.13 ± 0.32
DFE, mcg §	500.9*** ± 24.6	558.1*** ± 20.7	528.7*** ± 23.7	57.2 ± 34.9	27.8 ± 34.2	-29.3 ± 36.0
**Changes from baseline to 3mpp**						
	n=187	n=198	n=169			
Energy, kcal	-52.2 ± 58.6	-45.5 ± 69.5	-82.3 ± 86.8	6.8 ± 100.4	-30.0 ± 115.3	-36.8 ± 109.1
Carbohydrates, g	-23.8* ± 11.8	-21.9* ± 10.3	-23.7* ± 11.7	1.9 ± 14.3	0.1 ± 18.5	-1.8 ± 15.4
Protein, g	3.95 ± 2.29	-3.57 ± 2.75	1.08 ± 3.47	-7.52* ± 3.78	-2.87 ± 4.42	4.65 ± 4.36
Lipids, g	0.81 ± 4.02	2.20 ± 3.75	0.84 ± 4.26	1.39 ± 5.56	0.03 ± 6.14	-1.36 ± 5.90

Iron, mg	10.54*** ± 0.57	12.44*** ± 0.73	13.01*** ± 0.72	1.89 ± 1.02	2.47* ± 1.03	0.57 ± 0.97
Zinc, mg	11.30*** ± 0.62	12.69*** ± 0.55	13.45*** ± 0.68	1.39 ± 0.87	2.15* ± 0.89	0.76 ± 0.85
Vitamin C, mg	5.0 ± 10.1	29.1*** ± 8.6	30.1** ± 9.9	24.1 ± 14.8	25.1 ± 15.1	0.9 ± 12.8
Vitamin E, mg	6.12*** ± 0.95	8.34*** ± 0.55	7.73*** ± 0.99	2.23* ± 1.11	1.61 ± 1.42	-0.62 ± 1.20
Vitamin B12, mcg	1.75*** ± 0.27	2.02*** ± 0.30	2.17*** ± 0.32	0.27 ± 0.43	0.42 ± 0.45	0.15 ± 0.44
DFE, mcg §	461.3*** ± 30.4	524.6*** ± 36.6	556.0*** ± 44.6	63.4 ± 52.7	94.7 ± 56.5	31.4 ± 57.3

† Estimates are median changes or differences of median changes between study groups ± standard error

*p<0.05,**p<0.01,***p<0.001

‡ Beverage: Fortified beverage, Tablets: Micronutrient tablets and MNP: Micronutrient powder

Φ 25wp: 25 weeks of pregnant (baseline), 37wp: 37 weeks of pregnant, 1mpp: 1 month post-partum, 3 mpp: 3 months post-partum

§ DFE=Dietary folate equivalents (bioavailability= folic acid x 1.7)

Table S3. Covariate-adjusted median† energy intake of food groups from diet alone, by evaluation study groups ‡ and period $^{\Phi}$

		Beverage		Tablets		MNP	
		Median	(95% CI)	Median	(95% CI)	Median	(95% CI)
Dairy	37wk	158a	(128, 188)	192	(157, 227)	210	(172, 249)
	1mpp	196	(168, 224)	211	(184, 239)	242	(212, 271)
	3mpp	167	(141, 192)	172	(143, 202)	198	(166, 229)
Fast Food	37wk	145	(112, 177)	157	(136, 178)	133	(97, 168)
	1mpp	115	(89, 141)	132	(109, 155)	125	(97, 152)
	3mpp	164	(144, 184)	187	(161, 213)	162	(136, 187)
Leguminous	37wk	26	(19, 34)	20	(16, 24)	20	(15, 24)
	1mpp	23	(17, 29)	22	(16, 28)	19	(14, 24)
	3mpp	25	(19, 30)	22	(16, 29)	25	(19, 31)
Drinks	37wk	176	(136, 216)	188	(159, 217)	204	(172, 235)
	1mpp	128	(99, 157)	149	(120, 178)	149	(124, 174)
	3mpp	176	(152, 200)	163	(135, 191)	173	(153, 192)
Snack, Candies and Dessert	37wk	86	(61, 110)	91	(65, 117)	99	(86, 111)
	1mpp	57	(49, 66)	61	(50, 73)	55	(48, 63)
	3mpp	84	(64, 104)	78	(60, 96)	92	(73, 111)
Soups, cream and paste	37wk	30	(25, 35)	31	(27, 35)	29	(25, 33)
	1mpp	42	(36, 48)	47	(42, 52)	40	(35, 46)
	3mpp	34	(26, 42)	32	(25, 38)	34	(28, 40)
Fruits and vegetables	37wk	261	(221, 300)	279	(236, 323)	262	(231, 293)
	1mpp	123	(98, 147)	158	(135, 181)	172	(121, 223)
	3mpp	150	(137, 163)	**201b**	**(180, 223)**	159	(137, 182)
Meat,sausage and egg	37wk	162	(145, 179)	156	(136, 175)	155	(138, 172)
	1mpp	184	(162, 205)	207	(177, 236)	182	(158, 207)
	3mpp	185	(167, 203)	189	(167, 210)	180	(163, 198)
	37wk	1008	(877, 1140)	973	(833, 1113)	937	(810, 1064)

Cereals and root crop	1mpp	1109	(995, 1222)	1105	(986, 1224)	1096	(998, 1194)
	3mpp	1056	(910, 1201)	1001	(812, 1190)	1023	(896, 1151)

† Estimates are covariate-adjusted medians from a quantile regression that included school level, BMI, a wealth index and total energy at baseline as adjustment covariates. Standard errors were adjusted for data dependencies within communities.

‡ Beverage: Fortified beverage, Tablets: Micronutrient tablets and MNP: Micronutrient powder

Φ 37wp: 37 weeks of pregnant, 1mpp: 1 month post-partum, 3 mpp: 3 months post-partum

a p=0.042 MNP vs Beverage

b p=0.001 Tablets vs Beverage, p=0.010 Tablets vs MNP
